# Prognostic value of the distance between the primary tumor and brainstem in the patients with locally advanced nasopharyngeal carcinoma

**DOI:** 10.1186/s12885-016-2148-x

**Published:** 2016-02-17

**Authors:** Yuxiang He, Ying Wang, Lin Shen, Yajie Zhao, Pengfei Cao, Mingjun Lei, Dengming Chen, Tubao Yang, Liangfang Shen, Shousong Cao

**Affiliations:** Department of Oncology, Xiangya Hospital, Central South University, Hunan Province, No. 87, Xiangya Road, Changsha, Hunan Province 410008 PR China; School of Public Health, Central South University, Hunan Province, No. 87, Xiangya Road, Changsha, Hunan Province 410008 PR China; Xiangya Hospital, Central South University, Hunan Province, No. 87, Xiangya Road, Changsha, Hunan Province 410008 PR China

**Keywords:** Nasopharyngeal carcinoma, Intensity-modulated radiotherapy, Brainstem, Prognosis, Organs at risk

## Abstract

**Background:**

Brainstem dose limitations influence radiation dose reaching to tumor in the patients with locally-advanced nasopharyngeal cancer (NPC).

**Methods:**

A retrospective analysis of the prognostic value of the distance between the primary tumor and brainstem (Dbs) in 358 patients with locally-advanced NPC after intensity-modulated radiation therapy (IMRT). Receiver operating characteristic (ROC) curves were used to identify the cut-off value to analyze the impact of Dbs on tumor dose coverage and prognosis.

**Results:**

The three-year overall survival (OS), local relapse-free survival (LRFS), distant metastasis-free survival (DMFS), and disease-free survival (DFS) were 88.8 vs. 78.4 % (*P* = 0.007), 96.5 vs. 91.1 % (*P* = 0.018), 87.8 vs. 79.3 % (*P* = 0.067), and 84.1 vs. 69.6 % (*P* = 0.002) for the patients with the Dbs > 4.7 vs. ≤ 4.7 mm, respectively. ROC curves revealed Dbs (4.7 mm) combined with American Joint Committee on Cancer (AJCC) T classification had a significantly better prognostic value for OS (*P* < 0.05).

**Conclusions:**

Dbs (≤4.7 mm) is an independent negative prognostic factor for OS/LRFS/DFS and enhances the prognostic value of T classification in the patients with locally-advanced NPC.

## Background

The relationship of clear radiation dose–response has been confirmed for the patients with nasopharyngeal cancer (NPC). For example, Sze et al. [1] found that the risk of local failure increases by 1 % with every 1 cm increase in tumor volume. Additionally, Willner et al. [2] observed a dose–response relationship between the tumor volume and total radiation dose with regards to local control in the patients with NPC, and found that if the tumor volume doubled, an extra 5 Gy was required for achieving equivalent local control, and even a total dose of 72 Gy could not control the tumor with a volume larger than 64 ml. However, these studies were based on the patients with conventional radiotherapy.

A dose–response relationship still exists in the patients with NPC with intensity-modulated radiation therapy (IMRT), even though this new technique has significantly improved tumor dose coverage [3, 4]. However, Ng et al. [5] reported that the negative effect of the primary gross tumor volume (GTV_P) on local failure-free survival (LFFR) and disease-free survival (DFS) was outweighed by the volume of under-dosing due to neighboring neurological structures. In their analysis of 444 patients in whom dose tolerances were maintained for all critical neurological organs at risk (OARs), most patients with T4 disease (some with T3) were under-dosed (<66.5 Gy), and an under-dosed GTV_P volume of 3.4 cm^3^ was prognostic factor for poor LFFS and DFS. The volume of the GTV_P that is under-dosed (<66.5 Gy) is mainly affected by the neighboring neurological structures. Therefore, we hypothesize that the distance between the primary tumor and OARs may be a crucial factor for affecting survival outcomes in the patients with NPC.

Of all OARs that influence the tumor dose coverage, the brainstem is considered the most important factor, as brainstem dose restriction outweighs tumor dose coverage during the design of radiotherapy treatment plans. According to the Radiation Therapy Oncology Group (RTOG) 0225 [6] and 0615 [7] protocols, the ideal maximal point dose should be less than 54 Gy for the brainstem, and if the curative radiation dose cannot be achieved due to the brainstem dose tolerance, an acceptable alternative dose is <60 Gy to 1 % of the brainstem volume. However, in the patients with locally advanced NPC in whom the primary tumor is located close to the brainstem, the radical radiotherapy with IMRT cannot be delivered to some regions of the primary tumor. Ng et al. [5] reported that good target dose coverage could be achieved for the patients with T1-3 disease. However, under-dosed regions occurred in most patients with T4 disease, with an average volume of 3.4 cm^3^ of the primary tumor receiving <66.5 Gy (95 % of the prescribed dose of 70 Gy), and under-dosing of regions of the primary tumor close to the brainstem may account for the poor prognosis in the patients with T4 disease.

In the present study, we evaluated the impact of the distance between the primary tumor and brainstem (Dbs) on tumor dose coverage and investigated whether the Dbs is a potential prognostic factor in the patients with locally-advanced NPC receiving IMRT.

## Methods

### Patients

A total of 358 consecutive patients diagnosed with locally-advanced NPC (T3/T4N0-3M0) who received IMRT between August, 2008 and December, 2011 at Xiangya Hospital of Central South University (Changsha, Hunan province, China) were enrolled in this study. All patients were diagnosed via nasopharyngeal biopsy and nasopharyngeal and neck MRI examinations. In this study, 346 out of 358 patients were eligible for survival analysis due to the loss of 12 patients to follow-up. This study was approved by the ethics committee of Xiangya Hospital of Central South University (ID number: 2011 1086) and all participants have signed the informed consent form. The clinical characteristics of the patients are summarized in Table 1. The median age of the patients was 46 year-old (range, 17–82 years).

**Table 1:** The characteristics of the patients with locally-advanced nasopharyngeal carcinoma

Characteristic	Group	Number (%)		*P* value
		Dbs > 4.7 mm (*n* = 220)	Dbs ≤ 4.7 mm (*n* = 138)	
Age (years)	<50	143 (65.0)	89 (64.5)	0.922
	≥50	77 (35.0)	49 (35.5)	
Gender	Male	159 (72.3)	96 (69.6)	0.582
	Female	61 (27.7)	42 (30.4)	
T classification	T3	52 (23.6)	12 (8.7)	<0.001
	T4	168 (76.4)	126 (91.3)	
N classification	N0	44 (20.0)	22 (15.9)	0.514
	N1	69 (31.4)	44 (31.9)	
	N2	67 (30.5)	51 (37.0)	
	N3	40 (18.2)	21 (15.2)	
Histological type (WHO)	I	13 (5.9)	12 (8.7)	0.314
	II& III	207 (94.1)	126 (91.3)	
Chemotherapy	Yes	204 (92.7)	133 (96.4)	0.153
	No	16 (7.3)	5 (3.6)	
Prescribed dose	<73.92 Gy	74 (33.6)	37 (26.8)	0.174
	≥73.92 Gy	146 (66.4)	101 (73.2)	

### Clinical staging

In addition to CT/MRI examination of the nasopharynx and neck, the pre-treatment evaluation also included a complete medical history, physical examination, chest X-ray and/or CT (all patients with N3 disease underwent a chest CT), B-ultrasound scan of the abdomen and neck, bone scan and routine laboratory analysis. To reduce subjectivity, all patients were restaged according to the 7th edition of the American Joint Committee on Cancer (AJCC) Staging System for NPC; the MRI images for each patient were independently reviewed by two senior clinicians from the Departments of Radiology and Oncology.

### Definition of the Dbs

We re-contoured the brainstem for each patient according to its anatomic location on CT–MRI fusion images. The definition of brainstem-planning risk volume (PRV) included the brainstem plus a 1 mm margin. Measurement of the Dbs was performed as follows: the brainstem was serially extended by margins ranging from 1 to 10 mm to find the nearest point between the tumor and brainstem (to avoid visual inaccuracy), and the vertical distance between the closest edge of the primary tumor and the surface of the brainstem was measured; the distance was recorded as 10 mm as the maximal value even it exceeded 10 mm. Receiver operator characteristic (ROC) curve analysis was used to calculate the cut-off value for the Dbs with respect to overall survival (OS).

To determine the sacrificed volume of PGTVnx (SV-PGTVnx): firstly, we defined a new PGTVnx in order to eliminate the inconsistencies in expansion of the PGTV boundary between patients, which included the GTVnx and a 5 mm margin in all directions except for a 3 mm margin in the posterior direction. Secondly, the volume of overlap between the new PGTVnx and brainstem was calculated to obtain the SV-PGTVnx (ml). Thirdly, the values of radiation dose delivered in 1 cc (D1cc) and dose received by 1 % of the volume (D1 %) for the brainstem-PRV and the values of the maximum radiation dose (Dmax), the mean radiation dose (Dmean), and the minimum radiation dose (Dmin) of primary tumor, the dose covering the 95 % PTV (D95 %), the volume receiving the 95 % prescribed dose (V95 %) for the new PGTVnx were determined.

### Radiotherapy

All patients underwent IMRT. The target volumes were defined with reference to International Commission on Radiation Units and Measurements (ICRU) reports No. 50 and No. 62. The primary tumor (GTVnx) and positive lymph nodes (GTVnd) were defined; the retropharyngeal lymph nodes were included in the GTVnx. Two clinical target volumes (CTVs) were defined, as described in our previous study [8]. The corresponding planning target volumes (PTVs) were generated by extending each CTV by 3 mm; the prescribed doses for the PGTVnx (GTVnx + 3–5 mm margin) were 66.0–75.9 Gy; GTVnd, 69.96–72.6 Gy; PTV1, 59.4–64.0 Gy, and PTV2, 50.0–54.0 Gy. The doses to the PTV2 were administered over 28 fractions and other doses over 33 fractions. All patients were treated with simultaneous modulated accelerated radiotherapy once a day for 5 days a week. The dose limits for the critical normal tissue structures and plan evaluation were defined by Radiation Therapy Oncology Group (RTOG) protocol 0225 [6] and the dose constrains are as follows: brainstem, optic nerves, and chiasm ≤ 54 Gy or 1 % of the PTV ≤ 60 Gy; spinal cord ≤ 45 Gy or 1 cc of the PTV ≤ 50 Gy; temporal lobes ≤ 60 Gy or 1 % of the PTV ≤ 65 Gy. mandible and T-M joint ≤ 70 Gy or 1 cc of the PTV ≤ 75 Gy; tongue < 55 Gy or 1 % of the PTV ≤ 65 Gy; inner/middle ears mean dose < 50 Gy; glottic larynx mean dose < 45 Gy; parotid glands mean dose < 26 Gy (should be achieved in at least one gland) or at least 20 cc of the combined volume of both parotid glands < 20 Gy or at least 50 % of the gland < 30 Gy (should be achieved in at least one gland). Dose constraints for brainstem and spinal cord have the higher priority than GTV or CTV coverage while other normal structures will be considered lower priority than GTV or CTV coverage.

### Chemotherapy

Chemotherapy was part of the treatment plan for all patients except 21 patients who were unwilling to receive or could not tolerate chemotherapy. Neoadjuvant chemotherapy was administered when the waiting time for radiotherapy was longer than acceptable or to downsize bulky tumors. At the end of radiotherapy, adjuvant chemotherapy was administered to the patients with N2/N3 stage disease and the patients with existing residual disease detected by MRI or physical examination. Neoadjuvant chemotherapy or adjuvant chemotherapy consisted of cisplatin plus 5-fluorouracil or taxanes every 3 weeks for two or three cycles. Concurrent chemotherapy consisted of 80 mg/m^2^ cisplatin every 3 weeks. There were 193 patients received 2 cycles and 153 patients received more than 3 cycles of chemotherapy.

### Follow-up

The follow-up methods included direct telephone calls to the patients or their families; or hospital visits for the patients. Follow-up was measured from the first day of treatment to the last date of follow-up (January, 2015) or the date of death. After radiotherapy, follow-up examinations were conducted once every 3 months in the first 2 years, once every 6 months in years 2 to 5, and annually thereafter. Recurrence was defined as tumor recurrence after the tumor was undetectable for at least 1 month. The duration of OS was calculated from the day of radiotherapy completion to the date of death or last follow-up; LRFS, to the date of local recurrence; and DFS, to the date of tumor recurrence, distant metastasis or death.

### Statistical analysis

All statistical analyses were performed using Statistical Package for the Social Sciences version 17.0 (SPSS, Chicago, IL, USA). The Dbs data were subjected to normality testing; then Mann–Whitney tests of non-parametric data were used to analyze the relationships between the Dbs, T3/T4 disease, survival outcomes, GTVnx Dmin, V95 % and D95 % in the different groups. Actuarial rates were calculated using the Kaplan-Meier method and compared with the log-rank test. Multivariate analyses with the Cox proportional hazards model were used to test for independent significance by backward elimination of insignificant explanatory variables. ROC curve analysis was used to compare prognostic value. The criterion for statistical significance was set at α = 0.05 and all *P*-values were based on two-sided tests (two tailed).

## Results

### Treatment outcomes

The median follow-up period for all patients was 45 months (range, 3–78 months). The characteristics of the entire cohort of 358 patients with locally-advanced NPC are summarized in Table 1. In total, 22 out of 346 patients developed local recurrence (6.36 %), 55 out of 346 patients developed distant metastasis (15.9 %), and 9 out of 346 patients developed recurrence plus distant metastasis (2.6 %). There were 64 deaths among the 346 patients (18.5 %), of which 49 were due to tumor recurrence and metastasis, 10 were due to tumor-associated complications, one was due to gastrointestinal bleeding and four were due to unknown causes.

### The distribution of Dbs in the patients with locally-advanced NPC

**Fig. 1:**
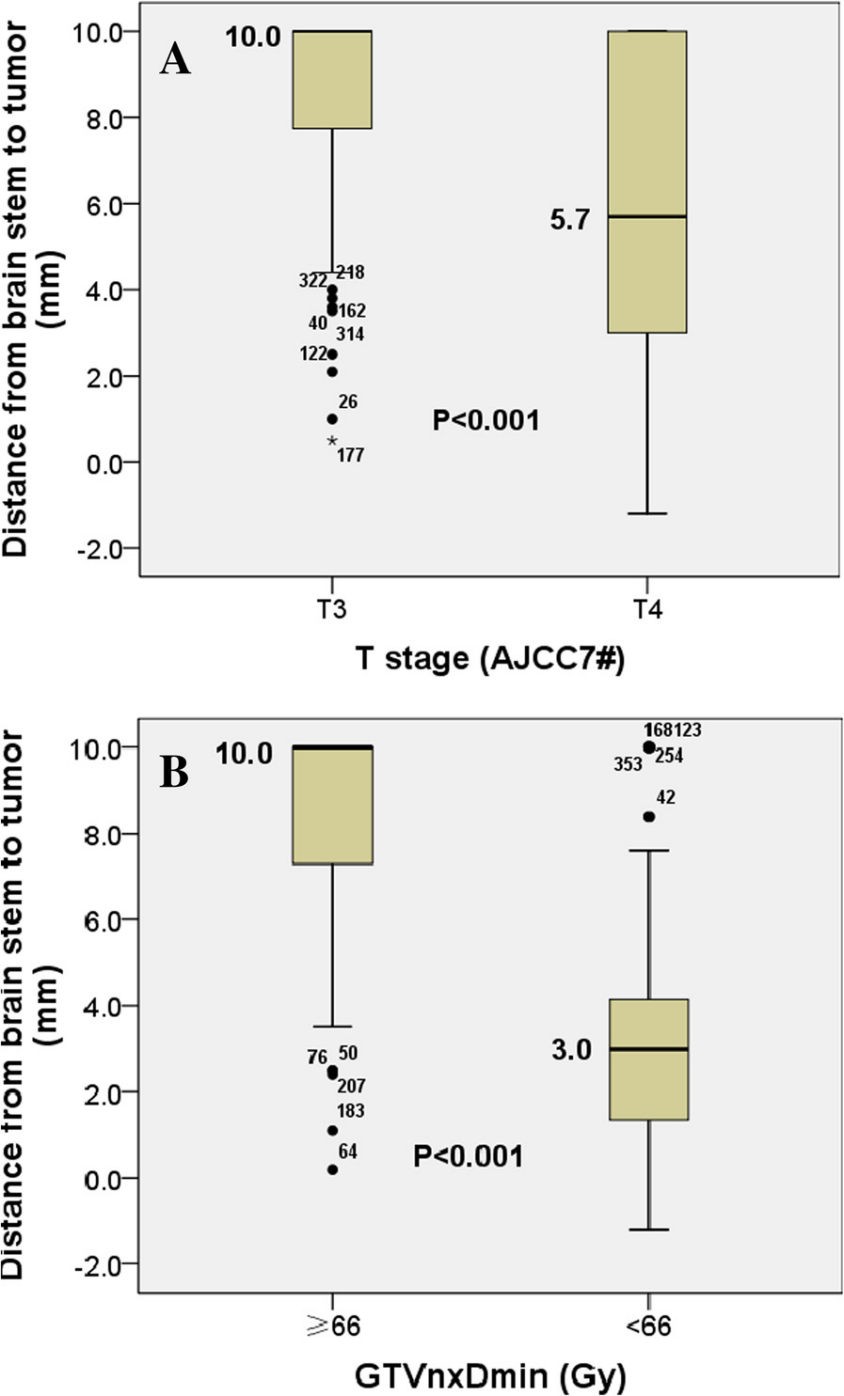
The distribution of the distance between the primary tumor and brainstem (Dbs) in the patients with locally-advanced nasopharyngeal carcinoma stratified by T classification (**a**) and GTVnx Dmin (**b**)

The overall distribution of Dbs in the patients with locally advanced NPC was a non-normal distribution (*P* > 0.10). As shown in Fig. 1a and Table 2, the median Dbs was 8.3 mm (range, 0.5 to 10 mm) in the patients with T3 disease and 5.7 mm (range, −1.2 to 10 mm) in the patients with T4 disease. The Mann–Whitney test suggested that the median Dbs was significantly lower in the patients with a T4 classification than the patients with T3 classification (*P* < 0.001). The median Dbs are 3.0 mm (range, −1.2 to 10 mm) in the patients of GTVnx Dmin < 66Gy and 8.6 mm (range, 0.5 to 10 mm) in the patients of GTVnx Dmin ≥ 66Gy (Fig. 1b and Table 2).

**Table 2:** The distance from the primary tumor to the brainstem (Dbs) in the patients with locally-advanced nasopharyngeal carcinoma

Dbs (mm)	T stage (7th AJCC)		GTVnx Dmin	
	T3 (*n* = 64)	T4 (*n* = 294)	≥66Gy	<66Gy
Mean ± SD	8.3 ± 2.7	5.94 ± 3.6	8.6 ± 0.15	3.0 ± 0.19
Median	10	5.7	10	3.0
Range	0.5–10	−1.2–10	0.2–10	−1.2–10

### A small Dbs is associated with a reduced dose to the primary tumor in the patients with locally-advanced NPC

**Fig. 2:**
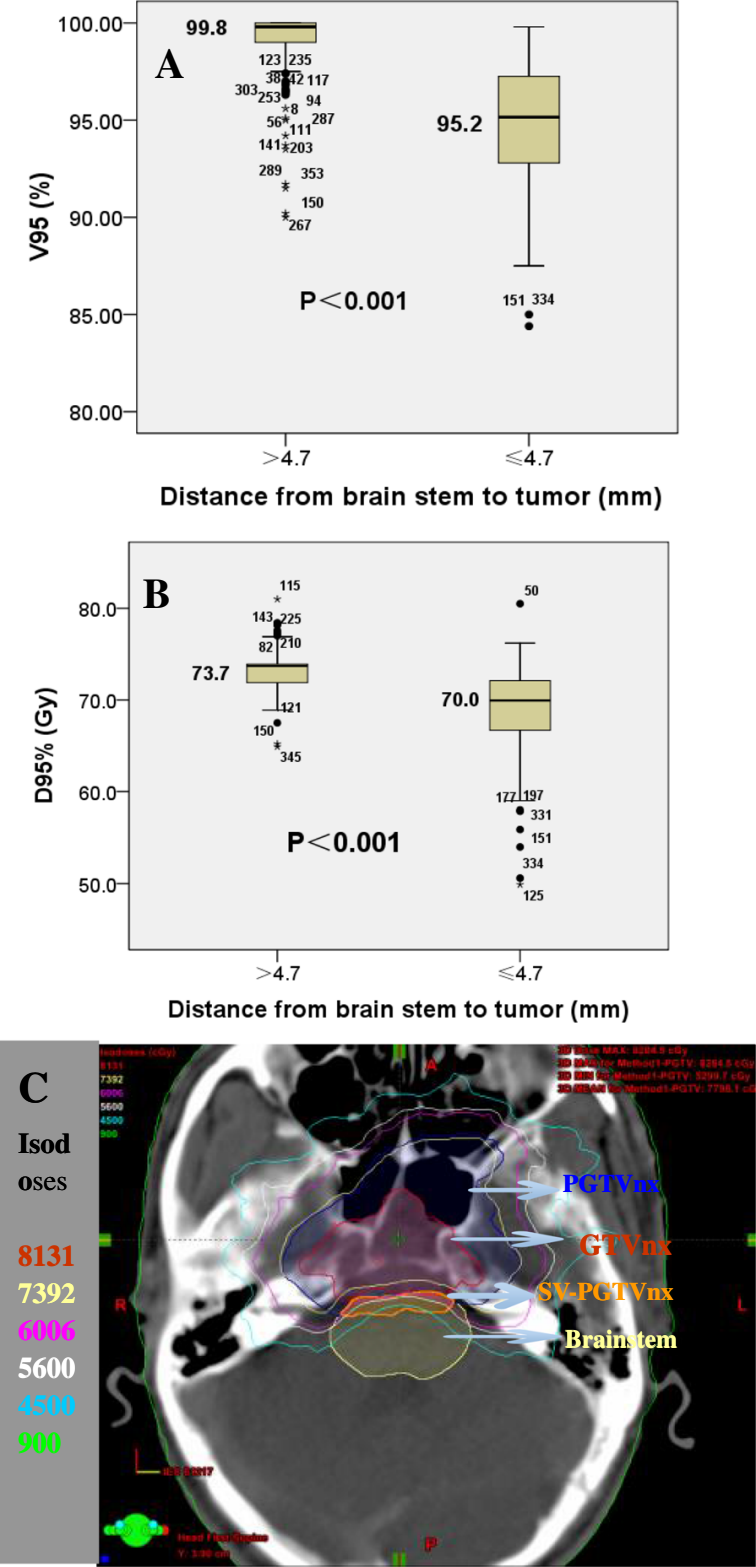
Relationship of the distance between the primary tumor and brainstem (Dbs) and PGTVnx V95 % (**a**); PGTVnx V95 % (**b**); and the radiation dose to GTVnx (**c**) of the patients with locally-advanced NPC. The color-filled areas represent the target volumes: GTVnx (*red*); PGTVnx (*blue*); brainstem (*yellow*); and sacrificed volume of PGTVnx (SV-PGTVnx, *orange*)

The patients were divided into two groups according to ROC analysis: Dbs ≤ 4.7 mm (220 patients) and Dbs > 4.7 mm (138 patients). The difference of prescribed radiation doses to the PGTVnx for the two groups was not statistically significant (73.6 vs 73.5 Gy, *P* > 0.05), however, the D95 % and V95 % values of the PGTVnx were significantly lower in the patients of Dbs ≤ 4.7 mm than in the patients of Dbs > 4.7 mm (median D95 %: 70.0 vs. 73.7 Gy, *P* < 0.001, and median V95 %: 95.2 vs. 99.8 %, *P* < 0.001, respectively), as shown in Fig. 2a and 2b and Table 3. These data indicate that the D95 % and V95 % decrease as the Dbs becomes smaller.

**Table 3:** D95 % and V95 % of the PGTVnx for the patients with locally-advanced nasopharyngeal carcinoma stratified by the distance from the primary tumor to the brain stem (Dbs)

D95 %/V95 %	D95 % (Gy)		Total	V95 % (%)		Total
	Dbs ≤ 4.7	Dbs > 4.7		Dbs ≤ 4.7	Dbs > 4.7	
Mean ± SD	69.0 ± 4.9	73.1 ± 2.0	71.5 ± 4.0	94.8 ± 3.2	99.1 ± 1.6	97.5 ± 3.2
Median	70.0	73.7	72.3	95.2	99.8	98.8
Range	49.9–80.5	64.9–81.0	49.9–81.0	84.4–99.8	90–100	84.4–100

As shown in Table 4, the patients with a smaller Dbs (≤4.7 mm) had a larger GTV-P, a larger SV- PGTVnx, and the lower values of Dmin, D95 %, and V95 % for the PGTVnx compared to the patients with a larger Dbs (>4.7 mm). However, the differences of these parameters were much smaller between the patients with T3 and the patients with T4 classifications. The data suggest that the Dbs has a greater influence on the smaller dose to the primary tumor than that of T classification. These radiation doses of Dmax, D1 %, D1cc, D1/3, Dmean were not significantly different for the brainstem between the patients with a small and large Dbs, nor with T3 and T4 classifications (Table 4).

**Table 4:** Radiation doses to the PGTVnx and brain stem for the patients with locally-advanced nasopharyngeal carcinoma stratified by T classification and the distance from the primary tumor to the brain stem (Dbs)

Mean (range)	Dbs ≤ 4.7 mm	Dbs > 4.7 mm	7th AJCC T3	7th AJCC T4
Tumor volume				
GTV-P (ml)	63.0 (16.5–204.8)	33.6 (5.9–164.0)	25.3 (6.3–73)	49.2 (5.9–204.8)
SV-PGTVnx (ml)	0.52 (0–12.4)	0.002 (0–0.2)	0.02 (0–0.44)	0.24 (0–12.4)
PGTVnx				
Dmin (Gy)	46.4 (21.9–66.6)	63.1 (21.7–74)	62.5 (38.6–73.6)	55.4 (21.7–74)
Dmean (Gy)	75.4 (65.6–83.9)	75.8 (67.6–84.1)	76.2 (71.0–81.1)	75.6 (65.6–84.1)
Dmax (Gy)	80.3 (69.5–88.7)	79.3 (70.9–88.8)	79.8 (76–84.1)	79.7 (69.5–88.8)
D95 % (Gy)	69.0 (49.9–80.5)	73.1 (64.9–81)	72.8 (57.9–78.4)	71.2 (49.9–81)
V95 % (Gy)	94.8 (84.4–98.8)	99.1 (90–100)	98.8 (90.2–100)	97.2 (84.4–100)
Brain stem				
Dmax (Gy)	54.1 (45.1–68.4)	51.8 (41.7–68.8)	50.9 (42.7–56.3)	52.5 (41.7–68.8)
D1 %	49.1 (38.8–59)	46.7 (34.8–58.6)	46.3 (34.8–50.6)	47.9 (36.9–59.9)
D1cc (Gy)	47.6 (36.7–56.7)	45.3 (35.4–52.4)	44.9 (35.4–49.8)	46.4 (35.7–56.7)
D1/3 (Gy)	39.9 (28.7–51.2)	37.2 (22.8–53.8)	37.4 (22.8–53.8)	38.4 (26.1–51.2)
Dmean (Gy)	36.4 (25.8–49.8)	31.6 (19.7–41.7)	31.6 (19.7–37.5)	33.8 (21.1–49.8)

SV-PGTVnx: sacrificed volume of the PGTVnx

The data in Fig. 2c illustrate the relationship between the Dbs and radiation dose to the primary tumor. When the tumor was near the brainstem, some of the PGTVnx and GTVnx laid outside of the 60 Gy isodose lines. The minimum radiation dose of SV-PGTVnx (the orange filled areas) was lower than 45 Gy.

### Prognostic value of the Dbs in the patients with locally-advanced NPC

**Fig. 3:**
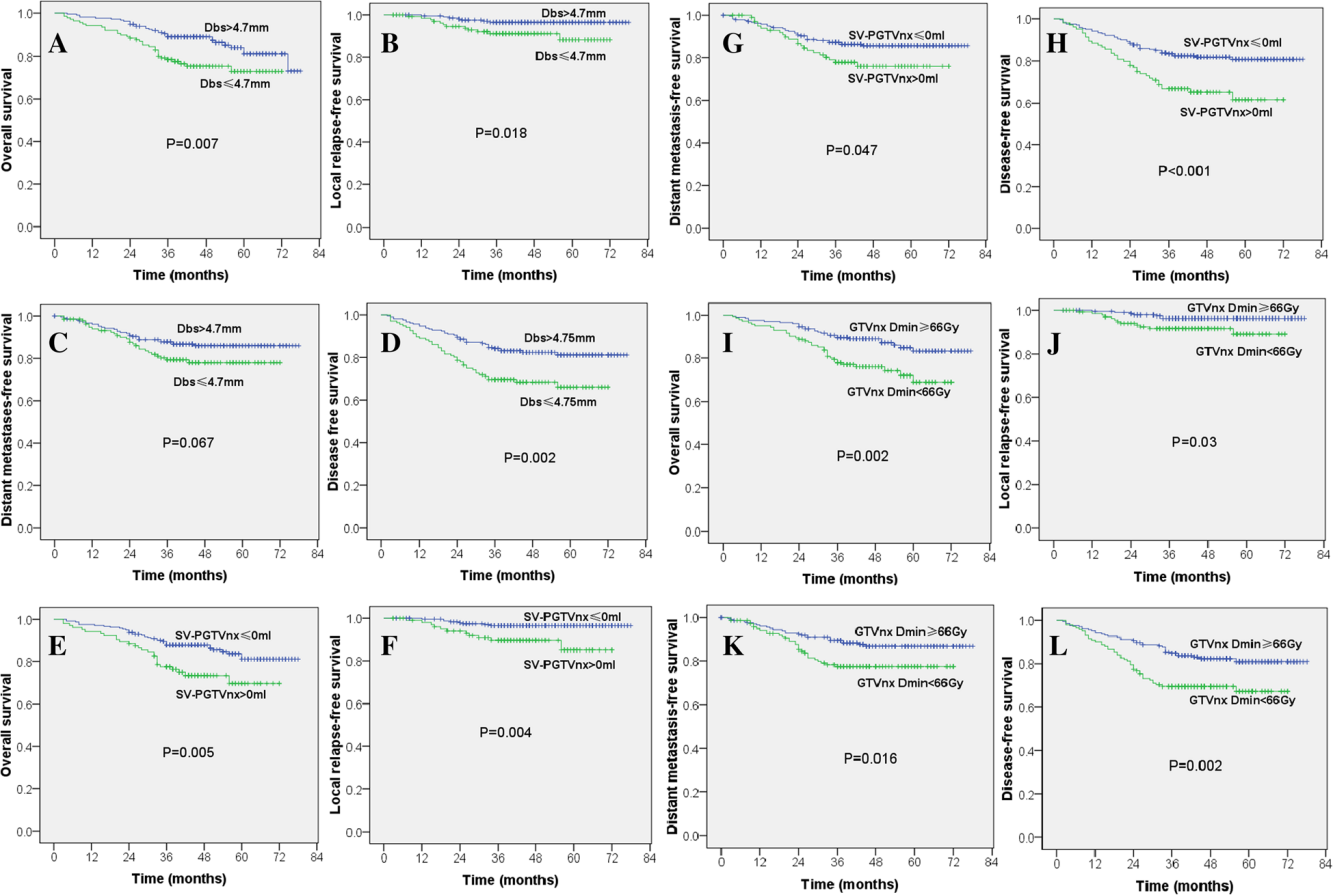
Survival curves of the patients with locally-advanced NPC stratified by the distance between the primary tumor and brainstem (Dbs > 4.7 mm or ≤ 4.7 mm, **a**-**d**); the sacrificed volume of PGTVnx (SV-PGTVnx ≤ 0 ml or > 0 ml, **e**-**h**); and the minimum radiation dose of primary tumor (GTVnx Dmin ≥ 66Gy or < 66 Gy, **i**-**l**)

The data in Fig. 3 show the survival curves of two groups of patients with different Dbs, SV-PGTVnx and GTVnx Dmin. The rates of 3-year OS, LRFS, DMFS and DFS for the two groups of patients stratified by Dbs (>4.7 mm or ≤ 4.7 mm) were 88.8 vs. 78.4 % (*P* = 0.007), 96.5 vs. 91.1 % (*P* = 0.018), 87.8 vs. 79.3 % (*P* = 0.067), and 84.1 vs. 69.6 % (*P* = 0.002), respectively. These were significantly different between the two groups, except DMFS (Fig. 3a-d, Table 5).

**Table 5:** Univariate analysis of prognostic factors in the patients with locally-advanced nasopharyngeal carcinoma receiving IMRT

Variable	No.# *N* = 346	3-year OS (%)	*p*-value	3-year LRFS (%)	*p*-value	3-year DMFS (%)	*p*-value	3-year DFS (%)	*p*-value
Age			0.003		<0.001		0.305		0.015
<50 y	225	87.8		97.7		86.6		82.1	
≥50 y	121	79.0		88.1		81.5		71.6	
N-stage			0.003		0.904		<0.001		0.018
N0	63	92.0		93.5		90.1		85.6	
N1	110	89.0		94		89.3		81.5	
N2	113	87.4		94.4		86.9		78.5	
N3	60	69.6		96.3		65.9		64.9	
T-stage			0.111		0.667		0.035		0.033
T3	59	89.6		93.2		93.2		88.1	
T4	287	83.7		94.7		82.8		76.4	
Overall stage			0.041		0.524		0.009		0.014
III	53	92.3		92.5		96.2		90.6	
IV	293	83.3		94.8		82.4		76.2	
Chemotherapy			0.386		0.242		0.921		0.374
Yes	327	85.1		94.8		84.7		79	
No	19	78.9		88.4		82.6		68.4	
Cycles of chemotherapy			0.841		0.853		0.835		0.988
<3	193	84.7		94.4		85.2		79.1	
≥3	153	84.8		94.5		87.0		77.6	
Prescribed dose			0.926		0.509		0.832		0.981
≤73.92 Gy	311	84.7		94.2		84.5		78.0	
>73.92 Gy	35	85.3		96.9		85.2		82.7	
GTVnx Dmin			0.002		0.03		0.016		0.002
<66 Gy	142	77.8		91.7		77.4		69.6	
≥66 Gy	204	89.6		96.3		89.4		84.6	
Dbs (mm)			0.007		0.018		0.067		0.002
≤4.7	136	78.4		91.1		79.3		69.6	
>4.7	210	88.8		96.5		87.8		84.1	

The rates of 3-year OS, LRFS, DMFS and DFS for the two groups of patients stratified by SV-PGTVnx (≤0 ml or > 0 ml) were 87.8 vs. 77.5 % (*P* = 0.005), 96.5 vs. 89.5 % (*P* = 0.004), 87.7 vs. 77.9 % (*P* = 0.047), and 83.3 vs. 66.9 % (*P* < 0.001), respectively. These were significantly different between the two groups (Fig. 3e-h, Table 5).

The rates of 3-year OS, LRFS, DMFS and DFS for the two groups of patients stratified by GTVnx Dmin (≥66Gy or < 66Gy) were 89.6 vs. 77.8 % (*P* = 0.002), 96.3 vs. 91.7 % (*P* = 0.03), 89.4 vs. 77.4 % (*P* = 0.016), and 84.6 vs. 69.5 % (*P* = 0.002), respectively. These were significantly different between the two groups (Fig. 3i-l, Table 5).

The univariate analysis suggests that the factors influencing the 3-year OS are age (*P* = 0.003), N-stage (*P* = 0.003), overall stage (*P* = 0.041), GTVnx Dmin (*P* = 0.002), and Dbs (*P* = 0.007), respectively. The factors influencing LFRS are age (*P* < 0.001), GTVnx Dmin (*P* = 0.003), and Dbs (*P* = 0.018), respectively. The factors influencing the 3-year DMFS are N-stage (*P* < 0.001), T-stage (*P* = 0.035), overall stage (*P* = 0.009), and GTVnx Dmin (*P* = 0.016), respectively. The factors influencing DFS are age (*P* = 0.015), N-stage (*P* = 0.018), T-stage (*P* = 0.033), overall stage (*P* = 0.014), GTVnx Dmin (*P* = 0.002), and Dbs (*P* = 0.002), respectively. However, chemotherapy and prescribed radiation dose are not the factors for significantly influencing the OS, LFRS, DMFS or DFS (Table 5).

The following parameters were included in the Cox proportional hazards model with backward elimination: Dbs (>4.7 vs. ≤ 4.7 mm), age (<50 vs. ≥ 50 years), gender (female vs. male), World Health Organization (WHO) histological grade (Type II & III vs. Type I), T classification (T3 vs. T4), N classification (N0 vs. N1 vs. N2 vs. N3), chemotherapy (with vs. without) and radiation doses (>73.92 vs. ≤ 73.92 Gy). As the results shown in Table 6, Dbs ≤ 4.7 mm was a negative independent prognostic factor for OS (HR = 1.929; *P* = 0.01), LRFS (HR = 2.84; *P* = 0.044), and DFS (HR = 1.977; *P* = 0.002), but not for DMFS (HR = 1.479; *P* = 0.156). Additionally, these parameters were identified as independent prognostic factors: age and N classification for OS; age for LRFS, N classification for DMFS; and age and N classification for DFS (Table 6).

**Table 6:** Multivariate analysis of prognostic factors in the patients with locally-advanced nasopharyngeal carcinoma receiving IMRT

End point	Variable	Regression coefficient	Standard error	*P*-value	Hazard ratio	95 % CI	
						Lower	Upper
OS	Age	0.845	0.252	0.001	2.328	1.422	3.812
	N classification	0.431	0.133	0.001	1.539	1.185	1.999
	Dbs	0.657	0.253	0.010	1.929	1.174	3.170
	T classification	0.383	0.438	0.381	1.467	0.622	3.460
LRFS	Age	1.802	0.578	0.002	6.061	1.953	18.804
	Dbs	1.044	0.518	0.044	2.840	1.030	7.831
	T classification	0.103	0.767	0.894	1.108	0.246	4.986
DMFS	N classification	0.451	0.145	0.002	1.570	1.182	2.085
	Dbs	0.391	0.276	0.156	1.479	0.842	6.638
	T classification	0.958	0.520	0.066	2.606	0.940	7.225
DFS	Age	0.600	0.225	0.008	1.822	1.172	2.835
	N classification	0.344	0.119	0.004	1.411	1.118	1.781
	Dbs	0.682	0.225	0.002	1.977	1.273	3.070
	T classification	0.576	0.403	0.153	1.780	0.808	3.920

Note: Disease staging was according to the 7th edition of the AJCC/UICC staging system

The following parameters were included in the Cox proportional hazards model with backward elimination: Dbs (>4.7 mm vs. ≤ 4.7 mm), age (<50 years vs. ≥ 50 years), gender (female vs. male), World Health Organization (WHO) histological grade (Type II & III vs. Type I), T classification (T3 vs. T4), N classification (N0 vs. N1 vs. N2 vs. N3), chemotherapy (with vs. without), and radiation doses (>73.92 Gy vs. ≤ 73.92 Gy)

### Predictive value of Dbs combined with T classification in the patients with locally-advanced NPC

**Fig. 4:**
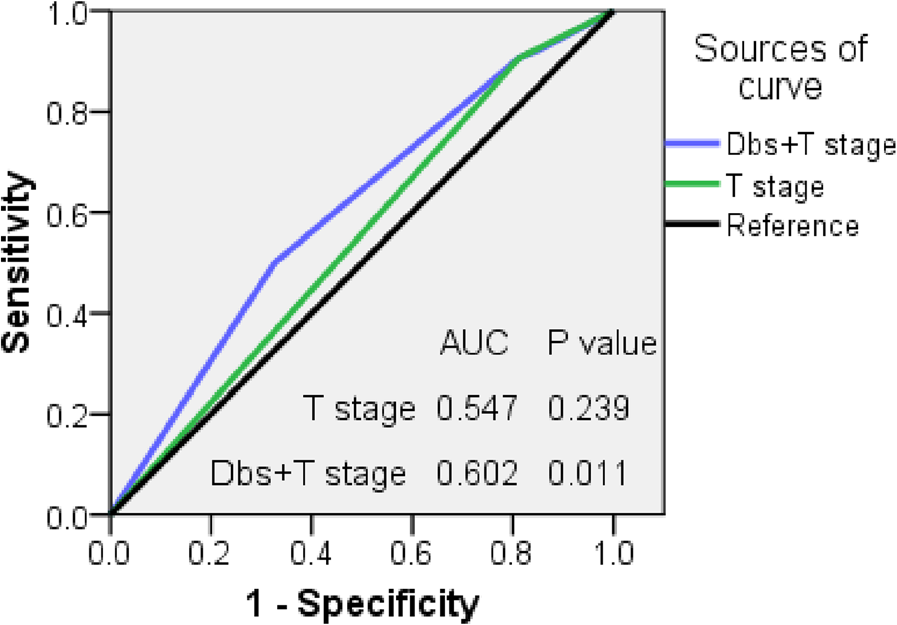
Receiver operator characteristic (ROC) curves of OS of the patients with locally-advanced NPC with T classification alone or in combination of T classification with Dbs

ROC curve analysis was used to assess the prognostic value of T classification alone or in combination of T classification with Dbs (Fig. 4). The combination of T classification with Dbs had a significant prognostic value for OS (AUC = 0.602; *P* = 0.011) but not with T classification alone (AUC = 0.547; *P* = 0.239).

## Discussion

The present study demonstrated that Dbs is an independent prognostic factor for OS, LRFS and DFS in the patients with locally-advanced NPC receiving IMRT, and the small Dbs hindered the improvement of the Dmin of tumor leading to poor prognosis. Compared to conventional radiotherapy, IMRT can improve target volume conformation while reducing the dose to the OARs. However, for the patients with locally-advanced NPC, the tumor lies close to the neighboring OARs, so it is difficult to achieve the desired dose distribution, even with IMRT [9]. In the study by Ng et al. [5], the average lowest dose for the GTV in T4 disease was 53.5 Gy (range, 29.9 to 70.1 Gy) and the average D95 % was 67 Gy (range, 55.5 to 71.4 Gy), which were far below the radical radiation dose required for T4 disease [2]. Chau et al. [10] analyzed IMRT dose distributions in the patients with T3-4 NPC, and reported the average Dmin to the GTV increased from 33.7 Gy for 2D-CRT to 62.6 Gy for IMRT, and the average D95 % increased from 57.1 Gy for 2D-CRT to 67 Gy for IMRT. However, it failed to achieve the tumor desirable doses and normal tissue dose limitations when the tumor was located close to OARs.

Abbasi et al. [11] identified the main factors affecting the tumor V95 % including advanced T classification, intracranial tumor invasion and a tumor volume greater than 200 cm^3^. The data in Fig. 2 and Tables 3 and 4 of the present study showed that at an equivalent prescribed dose, the values of average D95 %, V95 %, and PGTVnx Dmin were 70.0 Gy, 95.2 % and 46.4 Gy for the patients with a small Dbs (≤4.7 mm) and 73.8 Gy, 99.8 % and 63.1 Gy for the patients with a large Dbs (>4.7 mm), respectively. The data indicate that appropriate target dose coverage can be achieved in the patients with a large Dbs, but not in the patients with a small Dbs (Table 4). However, the median Dbs was only 3.0 mm in the patients with GTVnx Dmin < 66 Gy, which is much smaller than in the patients with GTVnx Dmin ≥ 66 Gy of 8.6 mm (Fig. 1b and Table 2). Therefore, the Dbs hinders further improvement in radiation dose for IMRT in the patients with locally-advanced NPC.

The reason for us to choose Dbs 4.7 mm as the cut-off value is from ROC curve analysis. The determination of ROC cut-off value is always complied with the principle of maximization in the sensitivity plus (1-specificity) or the maximization of the sum of the true positive rate and false negative rate, which is the optimal cut-off value. Therefore, we calculated the cut-off value of Dbs as 4.7 mm by ROC curve analysis. We initially investigated three distances: 1) Dbs > 5 mm ; 2) Dbs > 2 mm but  ≤ 5 mm; and 3) Dbs ≤ 2 mm before we chose ROC curve analysis to find the optimal cut-off value. The results showed that the 3-year OS, LRFS, DMFS, and DFS were 88.5 vs 85.6 vs 68.5 % (*P* = 0.003),96.2 vs 92.5 vs 88.9 % (*P* = 0.033), 87.5 vs 86.1 vs 68.7 % (*P* = 0.012), and 84.0 vs 78.6 vs 56.9 % (*P* < 0.001) for Dbs > 5 mm, Dbs > 2 mm but  ≤ 5 mm; and Dbs ≤ 2 mm, respectively. However, there are not the optimal cut-off values for comparison.

An insufficient dose of radiation is associated with reduced local control and a poor prognosis. For example, Ng et al. [5] reported that a bulky primary tumor was related to poorer OS; however, if a satisfactory dose of radiation (>70 Gy) was delivered to large tumors, the same treatment outcomes could be achieved similarly to small tumors. In addition, the effect of GTV_P volume on LFFR and DFS was outweighed by the degree of under-dosing. In our study, most patients had a prescribed dose of approximately 73.92 Gy and a D95 % of 70.22 Gy. The tumor volume under-dosed (<70.22 Gy) was mainly affected by the Dbs and limitation of the brainstem. Clinically, the dose priorities for the primary tumor and brainstem vary widely between different cancer centers and/or different physics technicians and oncologists. The Cancer hospital of Chinese Academy of Medical Sciences reported a brainstem Dmax of up to 80.3 Gy in the patients with T4 disease [12], and suggested that one possible strategy to treat advanced T4 disease is to drop the dose constraints for selected neurologic structures. However, the risk of radioactive brainstem injury was not mentioned in the report. Therefore, it is difficult to define the most appropriate dose tolerances for neurologic structures such as the brainstem, and long term follow-up studies are needed to monitor the complications caused by radiation. In the present study, the SV-PGTVnx and the Dbs were counterpart to an under-dosed volume as reported by Ng et al., but were more intuitively and conveniently due to the tumor closed to brainstem in the patients so the lower Dmin could be delivered into the tumor (Table 4).

To our knowledge, no study has been reported for Dbs affecting prognosis in the patients with NPC. In the present study, we had demonstrated that the 3-year OS, LRFS, DMFS and DFS was better for the patients with Dbs > 4.7 mm than the patients with Dbs ≤ 4.7 mm (*P* < 0.05 except for DMFS), and for the patients with SV-PGTVnx ≤ 0 ml than the patients with SV-PGTVnx > 0 ml (*P* < 0.05). The results are consistent with the report by Ng et al. [5], which they showed that the 5 year LRFS, DFS, and OS were 90.4 vs 54.3 %, 70.6 vs 26.0 %, and 76.8 vs 53.2 % (*p* < 0.001) for the patients with GTV-P 66.5 Gy < 3.4 cm3 and the patients with GTV-P 66.5  Gy ≥ 3.4 cm3, respectively. Their results indicate that the volume of tumor under-dosed (<66.5 Gy) had a significantly impact on the prognosis of the patients with NPC. The results suggest that the volume of tumor under-dosed (<66.5 Gy) was not only related to a short Dbs, but also to the dose tolerances for the optic nerve, optic chiasm and temporal lobe. Therefore, the exact tumor regions where insufficient dosing occurred are unknown. However, our study focused on the distance between the primary tumor and brainstem, as we followed the principle of prioritizing life-saving treatment during dose assessment, with priority was given to protection of the brainstem and spinal cord over treatment of the primary tumor, and the dose to the tumor outweighing the tolerances for the optic nerve, optic chiasm and temporal lobe. For advanced disease, we do not reduce radiation dose to tumor during treatment, even if the patient may bear an increased probability of radiotherapy-induced vision loss, blindness or temporal lobe damage. Based on such principle, only the brainstem and/or spinal cord are the key factor for the selection of radiation dose, while the optic nerve and others are less weighed compared to tumor treatment.

The reason for Dbs as an independent prognostic factor for OS, LRFS and DFS, but not for DMFS in the patients with locally-advanced NPC may be due to the fact that a short Dbs reduces local control, whereas DMFS is affected more by N classification, tumor volume, and biological characteristics of the tumor. Therefore, Dbs has less influence on DMFS than other parameters of survival outcomes.

SV-PGTVnx had a greater effect on the distribution of the isodose curves than that of Dbs. Moreover, we could not obtain the value of SV-PGTVnx in spite of Dbs can be obtained before treatment, which may affect us to make an appropriate decision when a patient needs an induction of chemotherapy to shrink the tumor in order to avoid brainstem injure. In the present study, we had demonstrates that the lower radiation dose of tumor had a worse treatment outcome (Fig. 3i-l) and a lower radiation doses may be due to a closer Dbs (Fig. 1b and Table 2). Moreover, age and N stage were independent prognostic factor for OS, LRFS and DFS (Tables 5 and 6). It had been proved that the older age had the worse prognosis and N staging affected the prognosis of the patients with NPC by Meta analyses [13, 14].

With advance treatment with comprehensive IMRT for the patients with NPC in recent years, the prognosis for the patients with locally-advanced NPC has been improved significantly, especially the local control rate. Many studies have found that the T classification had no predictive and prognostic values for local control and OS, whereas tumor volume was an important factor for prognosis in the patients with NPC [1, 2, 4, 15–20]. In the present study, the patients with a large Dbs also had a large primary tumor volume (GTV-P). As shown in Table 4, the mean volume of the GTV-P was 63.0 (16.5–204.8) ml for the patients with Dbs ≤ 4.7 mm and 33.6 (5.9–164.0) ml for the patients with the Dbs > 4.7 mm, respectively. In addition, Dbs affects the progression of patients with NPC mainly through lowering the radiation dose in the tumor and increasing the dose on the surrounding normal tissues. This is different with tumor volume affecting the progression of patients due to large tumor burden and increase of tumor Hypoxia.

Experience from the group of Hong Kong [21] indicated that dose escalation above 66 Gy in IMRT-based therapy was a significant determinant of progression-free survival and DMFS for the patients with an advanced T classification. This finding was also confirmed by our study (Fig. 3i-l). A small Dbs of patient is not only related to the bulky tumor volume, but also to lower delivered dose to the tumor, it is therefore bound to further influence prognosis. However, so far there is no report on how the distance between the primary tumor and OARs may affect prognosis. Our study has demonstrated that the Dbs is a very important independent prognostic factor in the patients with locally-advanced NPC. Additionally, the prognostic value significant improved when combined Dbs with T-stage (Fig. 4).

The limitations of our studies are the relatively short follow-up period in which radiation-induced brainstem injury and other late complications could not be assessed. Additionally, the number of cases is too small for T3 stage, and it’s a retrospective study. It is worth to further prospective study to elaborate the different effect of Dbs on the tumor dose and associated complications to find the appropriate individualized prescribed dose of future IMRT in the patients with NPC with different Dbs.

## Conclusions

In locally advanced NPC, Dbs (≤ 4.7 mm) is an independent negative prognostic factor for OS/LRFS/DFS and enhances the prognostic value of T classification. The findings may improve clinic stage of NPC and enable individualized cancer therapy according to the different tumor-brainstem distance.

## Abbreviations

AJCC: American Joint Committee on Cancer
CTVs: clinical target volumes
CI: confidence interval
DFS: disease-free survival
Dbs: distance between the primary tumor and brainstem
DMFS: distant metastasis-free survival
D95 %: dose covering the 95 % PTV
HR: hazard ratio
IMRT: intensity-modulated radiation therapy
ICRU: International Commission on Radiation Units and Measurements
LFFR: local failure-free survival
LRFS: local relapse-free survival
Dmax: maximum radiation dose
Dmean: mean radiation dose
Dmin: minimum radiation dose
NPC: nasopharyngeal cancer
OARs: organs at risk
OS: overall survival
PRV: planning risk volume
PTVs: planning target volumes
GTVnd: positive lymph nodes
GTVnx: primary tumor
GTV_P: primary tumor volume
RTOG: Radiation Therapy Oncology Group
ROC: receiver operating characteristic
SV-PGTVnx: sacrificed volume of PGTVnx
V95 %: volume receiving the 95 % prescribed dose
WHO: World Health Organization

## References

[1] Sze WM, Lee AW, Yau TK, Yeung RM, Lau KY, Leung SK (2004). Primary tumor volume of nasopharyngeal carcinoma: prognostic significance for local control. Int J Radiat Oncol Biol Phys.

[2] Willner J, Baier K, Pfreundner L, Flentje M (1999). Tumor volume and local control in primary radiotherapy of nasopharyngeal carcinoma. Acta Oncol.

[3] Zeng L, Tian YM, Sun XM, Huang Y, Chen CY, Han F (2014). Intensity-modulated radiotherapy for stage IVA/IVB nasopharyngeal carcinoma: clinical outcomes and patterns of failure in an endemic area in China. Strahlenther Onkol.

[4] Guo R, Sun Y, Yu XL, Yin WJ, Li WF, Chen YY (2012). Is primary tumor volume still a prognostic factor in intensity modulated radiation therapy for nasopharyngeal carcinoma?. Radiother Oncol.

[5] Ng WT, Lee MC, Chang AT, Chan OS, Chan LL, Cheung FY (2014). The impact of dosimetric inadequacy on treatment outcome of nasopharyngeal carcinoma with IMRT. Oral Oncol.

[6] Radiation Therapy Oncology Group RTOG protocol 0225: A phase II study of intensity modulated radiation therapy (IMRT) +/− chemotherapy for nasopharyngeal cancer. Available at: www.rtog.org. Last accessed date on October 24, 2015.

[7] RTOG 0615: A phase II study of concurrent chemoradiotherapy using three-dimensional conformal radiotherapy (3D-CRT) or intensity-modulated radiation therapy (IMRT) + bevacizumab (BV) [NSC 708865; IND 7921] for locally or regionally advanced nasopharyngeal cancer. Available at: www.rtog.org. Last accessed date on October 24, 2015.

[8] He Y, Zhou Q, Shen L, Zhao Y, Lei M, Wei R (2015). A retrospective study of the prognostic value of MRI-derived residual tumors at the end of intensity-modulated radiotherapy in 358 patients with locally-advanced nasopharyngeal carcinoma. Radiat Oncol.

[9] Kam MK, Chau RM, Suen J, Choi PH, Teo PM (2003). Intensity-modulated radiotherapy in nasopharyngeal carcinoma: dosimetric advantage over conventional plans and feasibility of dose escalation. Int J Radiat Oncol Biol Phys.

[10] Chau RM, Teo PM, Kam MK, Leung SF, Cheung KY, Chan AT (2007). Dosimetric comparison between 2-dimensional radiation therapy and intensity modulated radiation therapy in treatment of advanced T-stage nasopharyngeal carcinoma: to treat less or more in the planning organ-at-risk volume of the brainstem and spinal cord. Med Dosim.

[11] Abbasi AN, Hafiz A, Ali N, Khan KA (2013). Plan dose evaluation of three dimensional conformal radiotherapy planning (3D-CRT) of nasopharyngeal carcinoma (NPC): experience of a tertiary care University Hospital in Pakistan. Asian Pac J Cancer Prev.

[12] Cao CN, Luo JW, Gao L, Yi JL, Huang XD, Wang K (2013). Clinical outcomes and patterns of failure after intensity-modulated radiotherapy for T4 nasopharyngeal carcinoma. Oral Oncol.

[13] Pignon JP, le Maître A, Maillard E, Bourhis J, MACH-NC Collaborative Group (2009). Meta-analysis of chemotherapy in head and neck cancer (MACH-NC): an update on 93 randomised trials and 17,346 patients. Radiother Oncol.

[14] Zong J, Lin S, Lin J, Tang L, Chen B, Zhang M (2015). Impact of intensity-modulated radiotherapy on nasopharyngeal carcinoma: Validation of the 7th edition AJCC staging system. Oral Oncol.

[15] Feng M, Wang W, Fan Z, Fu B, Li J, Zhang S (2013). Tumor volume is an independent prognostic indicator of local control in nasopharyngeal carcinoma patients treated with intensity-modulated radiotherapy. Radiat Oncol.

[16] Chen C, Fei Z, Pan J, Bai P, Chen L (2011). Significance of primary tumor volume and T-stage on prognosis in nasopharyngeal carcinoma treated with intensity-modulated radiation therapy. Jpn J Clin Oncol.

[17] Kuang WL, Zhou Q, Shen LF (2012). Outcomes and prognostic factors of conformal radiotherapy versus intensity-modulated radiotherapy for nasopharyngeal carcinoma. Clin Transl Oncol.

[18] Lee CC, Ho HC, Lee MS, Hsiao SH, Hwang JH, Hung SK (2008). Primary tumor volume of nasopharyngeal carcinoma: Significance for survival. Auris Nasus Larynx.

[19] Wu Z, Su Y, Zeng RF, Gu MF, Huang SM (2014). Prognostic value of tumor volume for patients with nasopharyngeal carcinoma treated with concurrent chemotherapy and intensity-modulated radiotherapy. J Cancer Res Clin Oncol.

[20] Chen MK, Chen TH, Liu JP, Chang CC, Chie WC (2004). Better prediction of prognosis for patients with nasopharyngeal carcinoma using primary tumor volume. Cancer.

[21] Kam MK, Teo PM, Chau RM, Cheung KY, Choi PH, Kwan WH (2004). Treatment of nasopharyngeal carcinoma with intensity-modulated radiotherapy: the Hong Kong experience. Int J Radiat Oncol Biol Phys.

